# Celiac Disease Presenting with Immune Thrombocytopenic Purpura

**DOI:** 10.1155/2017/6341321

**Published:** 2017-03-23

**Authors:** Hakan Sarbay, Billur Cosan Sarbay, Mehmet Akın, Halil Kocamaz, Mahya Sultan Tosun

**Affiliations:** ^1^Pediatric Hematology and Oncology, Pamukkale University Faculty of Medicine, Denizli, Turkey; ^2^Pathology, Denizli State Hospital, Denizli, Turkey; ^3^Pediatric Gastroenterology, Pamukkale University Faculty of Medicine, Denizli, Turkey; ^4^Pediatric Gastroenterology, Denizli State Hospital, Denizli, Turkey

## Abstract

Celiac disease (CD) is an immunological disorder. Clinical manifestations occur as a result of intestinal mucosa damage and malabsorption. CD is also associated with extraintestinal manifestations and autoimmune disorders. The coexistence of CD and autoimmune diseases has been described before. In this article, a patient with CD presenting with thrombocytopenia is discussed.

## 1. Introduction

Celiac disease (CD) is an immunological disorder caused by gluten intolerance. Short stature and chronic diarrhea are the most common findings in CD [1, 2]. In addition to typical malabsorption symptoms, extraintestinal manifestations like skin lesions, osteoporosis, elevated transaminases, and hematological manifestations can be first findings of CD [2, 3]. Also, CD can be associated with autoimmune disorders [4]. In this article, a patient with CD presenting with thrombocytopenia is discussed.

## 2. Case

A 9-year-old girl was admitted to our pediatric hematology unit with petechiae, ecchymoses, and history of a viral illness. Physical examination revealed mild hepatomegaly, growth failure, and common petechiae and ecchymoses. Cardiovascular, pulmonary, and neurological examination were normal. There was no evidence of any preceding medication.

The initial investigations revealed a white blood cell count of 6360/mm^3^, hemoglobin of 11,4 gr/dl, and a platelet count of 28.000/mm^3^. No blast and average of 1-2 large platelets were seen in the blood smear. Further laboratory evaluation showed a prothrombin time of 12.1 s, an INR of 1.1, a partial thromboplastin time of 28 s, aspartate transaminase (AST) of 174 IU/L, alanine transaminase (ALT) of 193 IU/L, lactate dehydrogenase (LDH) of 914 U/L, and an erythrocyte sedimentation rate of 14/h; urea (22 mg/dL), creatinine (0.3 mg/dL), B12, and folic acid levels were normal. No evidence of viral infections—including the hepatitis A virus, the hepatitis B virus, the hepatitis C virus, the Epstein-Barr virus, the Rubella virus, and Cytomegalovirus—was found. Anti-nuclear antibody (ANA) was negative. Bone marrow aspiration smear that showed increased mature and immature megakaryocytes was consistent with ITP.

**Figure 1:**
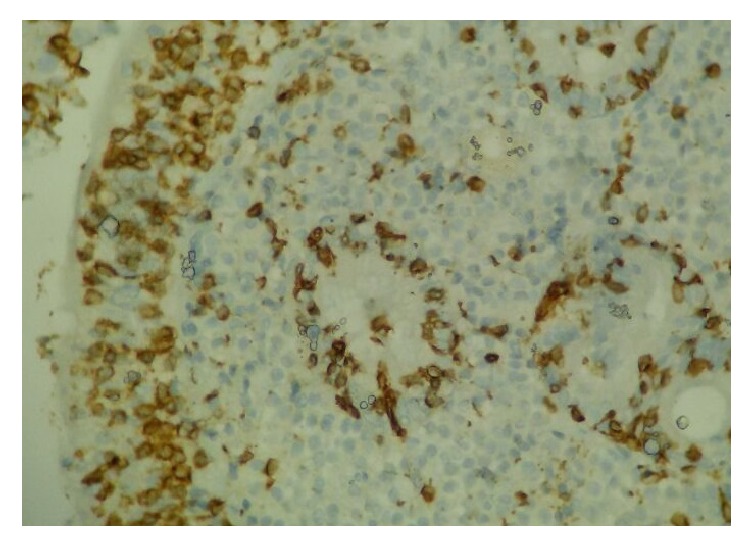
Total villous atrophy, increased number of intraepithelial lymphocytes, and crypt hyperplasia; CD3 ×400.

**Figure 2:**
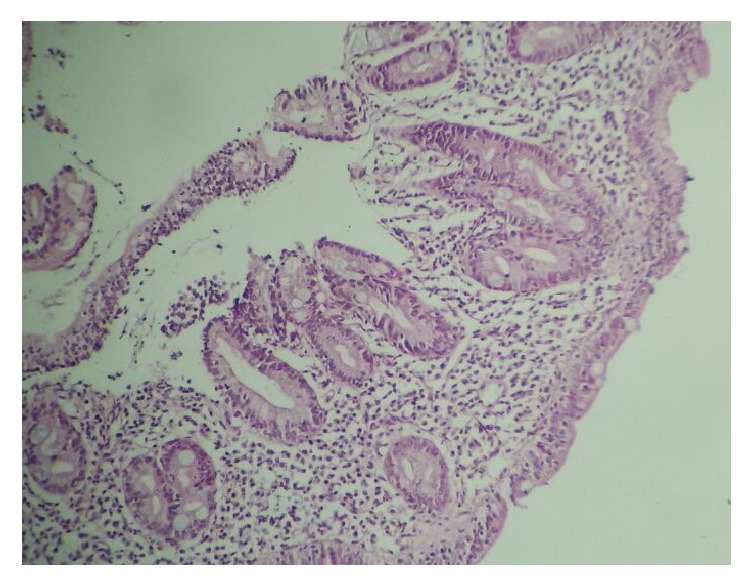
Total villous atrophy, increased number of intraepithelial lymphocytes, and crypt hyperplasia; Hematoxylin & Eosin ×100.

Tissue transglutaminase (TG) and IgA endomysium-specific antibodies (EMAs) were evaluated for the CD because of the growth failure and elevated transaminase levels. Tests showed positive values for CD. Therefore, an endoscopic biopsy was performed, and it was consistent with total villous atrophy, increased number of intraepithelial lymphocytes, and crypt hyperplasia in the intestinal mucosa (Figures 1 and 2).

After a month starting a gluten-free diet, platelet count increased to 87.000/mm^3^. Platelet count showed normal values (183.000/mm^3^) in the fifth month of the diet (Table 1). Any treatment for ITP was not given, such as immune globulin and steroids.

## 3. Discussion

Clinical manifestations occur as a result of intestinal mucosa damage and malabsorption in CD [5]. Iron deficiency is the most common hematological disorder because of the poor absorption of iron, and it is also one of the atypical symptoms of CD [5, 6]. Folic acid and B12 deficiencies can be seen, except regarding iron deficiencies [5]. In a study of 22 patients with CD, iron deficiency anemia (IDA) was detected in 21 patients, and also B12 deficiency was found in 7 of these patients. One patient had a folic acid deficiency in addition to B12 and iron deficiencies [7]. Leukopenia and thrombocytopenia can be also seen because of B12 and folic acid deficiencies; however autoimmune cytopenias are some of the clinical presentations of CD that develop with different autoimmune mechanisms [6].

The first case report of the coexistence of ITP and CD in a child was described in 1988 [8]. Numerous cooccurrences of CD and ITP have been widely described in literature, and it was showed that both diseases had similar autoimmune mechanisms [9]. The innate immune system plays an important role in the pathogenesis of CD, and Toll-like receptors (TLRs) are key players in the innate immune system [10]. Zanoni et al. [11] reported that patients with CD have a subset of transglutaminase antibodies that activate TLR4. In addition, it is thought that the TLR4 expression in platelets seems to be a prerequisite for thrombocytopenia [12].

Iron, ferritin, iron binding capacity, B12, and folic acid levels were within normal limits in our patient. The diagnosis of ITP was made because of the history of respiratory tract infection, isolated thrombocytopenia, and increased immature–mature megakaryocytes in the bone marrow. The initial investigations for CD revealed positive values for TG and EMAs, and intestinal biopsy showed total villous atrophy associated with an increased number of intraepithelial lymphocytes and crypt hyperplasia in the mucosa consistent with a diagnosis of CD.

The literature described how a gluten-free diet could improve ITP in a year without using corticosteroids or immunoglobulin [13]. After the administration of a gluten-free diet, the platelet count normalized in 5 months in our patient. On the other hand, the coincidences of undiagnosed CD with ITP are considered to be induced by viral infection, and even the platelet count increased independently from the gluten-free diet. However, our clinical experiences and reports in the literature of association between the two conditions suggest that diet plays an important role in treatment.

In conclusion, hematological abnormalities are frequently present in CD. Also, ITP is one of the atypical presentations of CD because of the similar autoimmune mechanisms. It is important that CD treatment be considered in the differential diagnosis of children who present with ITP.

## Figures and Tables

**Table 1:** Platelet count by months after gluten-free diet.

Month	1	2	3	4	5	7	9
Platelet count	87.000/mm^3^	95.000/mm^3^	112.000/mm^3^	137.000/mm^3^	183.000/mm^3^	168.000/mm^3^	199.000/mm^3^
